# Annotations

**Published:** 1905-06-24

**Authors:** 


### ANNOTATIONS.

### Direct Representatives.

The debate during the last session of the General Medical Council on the proposal of Mr. Geo. Brown to represent to the Privy Council the advisability of increasing the number of members of the Council elected by the votes of the profession was one of much interest. As was pointed out by Mr. Brown, there are at least two facts which afford a *primâ facie* case in favour of such increase. The first is that since 1886, when direct representatives first came into existence, the number of medical practitioners has considerably increased. In the second place, the institution of new universities in England, each of which sends a member to the Council, has raised the total membership of the Council from 30 to 33, thus involving a proportionate diminution of the extent of direct representation. Though the motion was lost by a majority of 18 to 10, the debate does not reveal any argument against the conclusion indicated by the facts just stated. The speeches in opposition were mainly occupied by considerations which, if valid, negative the existence of direct representation in any measure, and by protests that members sent to the Council by the various universities and corporations were really representative of professional interests in general, and not of the special interests of their constituencies. Such arguments are a little late in the day, and have a somewhat musty smell. It may be doubted whether standing alone they carry much force even with their sponsors. To say the Council is already too large is in the circumstances a more intelligible claim; but the answer of course is that other interests, and not merely that of direct representation, should be reduced. One speaker made effective use of the small percentage of the profession—roughly not more than 50 per cent.—taking part in the election of the direct representatives. This, to his mind, was conclusive against the view that the general body of the profession took much interest in the proposal. He therefore declined to vote, and four other members also abstained. Obviously this criticism has some force; and practitioners interested in the question might well stir up their friends to greater electoral activity.

### Nature and Man.

In his Romanes lecture Dr. Ray Lankester selected a topic closely allied to the one which on a similar occasion the late Professor Huxley made famous, and from a text which at first sight may appear of the speculative order he drew a number of severely practical conclusions. Whatever may have been the origin of the human mind it must, so the lecturer urged, be recognised as the one and all-powerful instrument with which man had contended, and was destined hereafter to contend, against extra-human nature. Hence the importance to human progress of a knowledge of the true relation of nature to man, and of the study of such questions as the hereditary transmission and conditions of development of mental qualities. Experimental psychology, though at present only in its infancy, was a necessity if man is to learn how to make the best use of the only weapon at his disposal in his struggle to control nature. Especially is knowledge of this order important to those destined to take an active part in the public affairs of the nation, and to act as the leaders of the great professions. Such considerations make it necessary to inquire whether in the existing educational scheme the truth of these propositions is recognised. And here Professor Lankester does not hesitate to speak in very plain terms. The University of Oxford he describes as exercising an injurious influence on education, and particularly on the education of those who will in coming years determine the action and opinion of the nation. For the present classical and historical method he would substitute, as the chief subject of education both in school and college, a knowledge of nature as set forth in the sciences of physics, chemistry, geology, and botany. The strongest inducements in the way of reward ought to be attached to progress in nature knowledge. In short, what is usually regarded as science is advanced both as the best instrument in the education of the individual and the essential condition for the progress and salvation of the community. These are certainly courageous notes in a university which only recently again affirmed its demand for Greek as an essential preliminary to academic studies. The lecturer himself recognises that in the meantime his proposals have no chance of acceptance. But to "tell" the dream may be an influence helping it to "come true."

### The Rich and the Birth Rate.

There is food for reflection in the return which has just been issued by the medical officer of health for Kensington. It is not a matter of any particular significance that the females in the Court suburb are greatly in excess of the males. This has for many years been the case, and it is partly due to the large percentage of females employed in domestic service. But the contrast presented between the birth rate of Kensington and that of the metropolis as a whole is noteworthy. Thus, the average birth rate of the capital last year was 27·9 per 1,000 of the population, and that of Kensington was only 19·3. The difference, moreover, is accentuated by the fact that, as Dr. Dudfield points out, the marriage rate in Kensington is considerably above the average in London and the country generally. Bearing in mind that Kensington is, *par excellence*, a rich parish, with many residents of great wealth, a considerable proportion of people of ample means, and a still greater one of persons in comfortable circumstances, the figures are distinctly disquieting. Do they portend, as some are afraid, a growing tendency on the part of men and women who are lavishly, or adequately, provided with this world's goods to limit their parental obligations to the utmost possible extent, to get as much pleasure out of life as they can with the minimum of anxiety and trouble? The prospect of a general decline of the birth-rate among the rich and well-to-do should suffice to excite serious misgivings in the minds of all who have at heart the best interests of the British nation. We hope that the low rate in the Court suburb is merely a passing incident and not a symptom of an increasing inclination on the part of the rich and prosperous to regard the bringing up of children as a burden to be shirked rather than as a duty to the State.

